# Intracochlear Pressure Changes due to Round Window Opening: A Model Experiment

**DOI:** 10.1155/2014/341075

**Published:** 2014-05-22

**Authors:** P. Mittmann, A. Ernst, I. Todt

**Affiliations:** Department of Otolaryngology, Head and Neck Surgery, Unfallkrankenhaus Berlin, Warenerstraße 7, 12683 Berlin, Germany

## Abstract

To preserve residual hearing in cochlea implantation, the electrode design has been refined and an atraumatic insertion of the cochlea electrode has become one aspect of cochlea implant research. The opening of the round window can be assumed to be a contributing factor in an atraumatic concept. The aim of our study was to observe intracochlear pressure changes due to different opening conditions of an artificial round window membrane. The experiments were performed in an artificial cochlea model. A round window was simulated with a polythene foil and a pressure sensor was placed in the helicotrema area to monitor intraluminal pressure changes. Openings of the artificial round window membrane were performed using different ways. Opening the artificial round window mechanically showed a biphasic behaviour of pressure change. Laser openings showed a unidirectional pressure change. The lowest pressure changes were observed when opening the artificial round window membrane using a diode laser. The highest pressure changes were seen when using a needle. The openings with the CO_2_ laser showed a negative intracochlear pressure and a loss of fluid. In our model experiments, we could prove that the opening of the artificial round window membrane causes various intracochlear pressure changes.

## 1. Introduction

The indication criteria for cochlea implantation have changed over the past decades to patients with residual hearing. As the criteria for cochlea implantation evolved, the perioperative surroundings, electrode design, and surgical technique have developed as well. Common surgical sense is that a selective scala tympani insertion should be achieved [1] and the insertion trauma should be minimised [2–6]. How to achieve the goal of reproducible preserved residual hearing is a matter of discussion. The insertion trauma is assumed to depend on different factors, and the contribution of each factor is unclear to some degree: the way and degree of opening the cochlea, insertion of the electrode array, additional medication (e.g., steroids and the form of application) [7], sealing of the cochlea, and the electrode design. The two major surgical techniques for access to the cochlea have been the round window approach and the “soft surgery” cochleostomy [8]. Following the pathway of a pure round window insertion, there are, to our knowledge, no studies that compare or describe the opening of the round window membrane. The anatomical conditions make it mandatory to drill away the promontorial lip to overlook the round window membrane [9], but the opening procedure itself has not been further evaluated.

There are different ways to open the cochlea. Opening of the round window membrane can be performed with blunt or sharp tools or with a laser. The cochlea is a fluid dynamic system. If the round window membrane is manipulated, the pressure is directly transferred into the cochlea and damage related to this force could occur. The aim of the present study was to compare different opening techniques with regard to pressure changes in a cochlea model.

## 2. Material and Methods

### 2.1. Pressure Sensor

The intracochlear pressure was measured using the microoptical pressure sensor developed by Olsen [10]. Details about design, fabrication, and capacity can be found in the literature [10]. Basically, the tip of the pressure sensor is a hollow glass tube sealed on one end by a plastic thin film diaphragm coated with a reflective surface of evaporated gold. The optical fibre is located in the glass tube with a small distance (50–100 *μ*m) to the diaphragm tip. The optical fibre is attached to a light-emitting diode (LED) light source and to a photodiode sensor. Light from the LED source reaches the sensor tip of the optical fibre, fans out as it exits the fibre, and is reflected by the gold-covered flexible diaphragm. The reflected light is sensed by the photodiode. A small pressure induces distance displacements of the diaphragm, which modulate the intensity of the reflected light. Time sensitivity of the sensor is 300 measurements per second. The sensor is connected to a module, which is again linked to a computer. Evolution software was used to record the intracochlear pressure.

### 2.2. Preparation of the Cochlea Model

**Figure 1:**
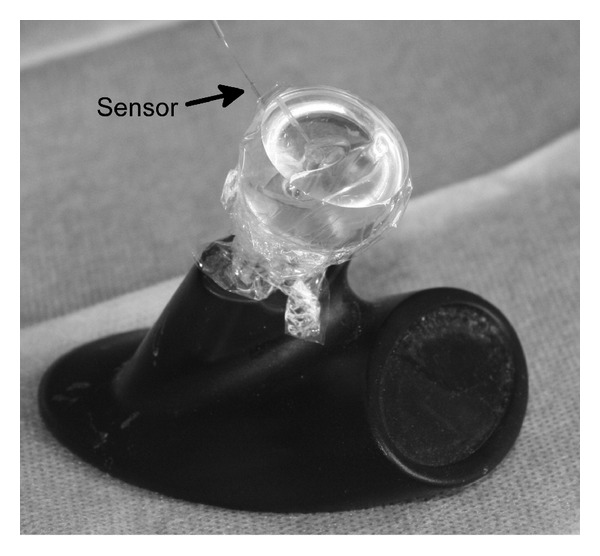
Artificial cochlea model with polythene foil. The sensor is placed in the helicotrema area.

**Figure 2:**
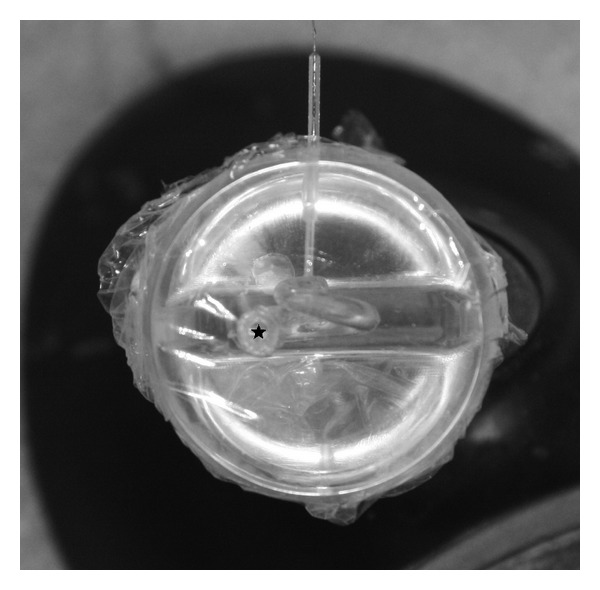
Top view on the cochlea model; the ★ marks the round window.

The experiments were performed in a synthetic transparent artificial cochlea model (Figure 1). The round window was a circular opening with a diameter of 1.5 mm and impressed slightly greater in comparison with other studies (1.23 mm) [11] (Figure 2). In the helicotrema area of the cochlea model, an extra channel was drilled that was slightly larger (about 800 *μ*m) than the sensor tip to insert the pressure sensor. After the pressure sensor was inserted, the cochlea was filled up with water and the position of the sensor within the channel was fixed and sealed with fibrin glue. The sensor was placed within the channel in such a way that the tip had contact to the edge of neither the channel nor the ground. The round window was covered with a commercially available polythene foil. Polythene foil was chosen because it is similar in its lack of resistance and distension and in tear strength to the natural round window membrane. Afterwards, the cochlea was microscopically controlled to suspend any enclosed air bubbles [12].

### 2.3. Measurements

The experimental setup was the same in every measurement. To standardise the conditions, all openings of the artificial round window membrane were performed by the same surgeon. The sensor was calibrated in the cochlea and the initial value was set to zero. A measurement was considered to be useful if the measured mmHg value after finalisation of the experiment was close to zero. Under these conditions, five openings of the round window membrane were performed. After every opening, the membrane was replaced by a new foil and the cochlea was refilled with water and checked microscopically for any enclosed air bubbles. We performed different opening procedures using a needle, a cannula, a diode laser, and a CO_2_ laser.

When opening the round window membrane with a needle, the needle was gently placed on the membrane and was pulled sideways carefully to perforate the membrane. A cannula was used to incise the membrane and then pulled sideways carefully to open the artificial membrane horizontally. With the diode laser, the tip was placed in the middle of the round window until a circular perforation of the round window membrane was seen under the microscope. Measurements were performed with different intensities using 4, 6, and 10 W. The opening of the round window membrane with the CO_2_ laser was performed with a Zeiss S5, OPMI TwinER (20 W). The finder sight was aimed at the centre of the round window membrane and several impulses were emitted until the membrane was perforated.

Since internationally different pressure sizes are used, a conversion table is attached: 1 mm/Hg = 133 pascal = 0.019 psi = 1.35 cm H_2_O.

## 3. Results

An opening of the artificial round window membrane was achieved in all attempts with that amount, which would allow an insertion of a CI electrode. With every tool, five openings of the round window membrane were performed. Changes of the intracochlear pressure were measured in mmHg (Table 1).

**Figure 3:**
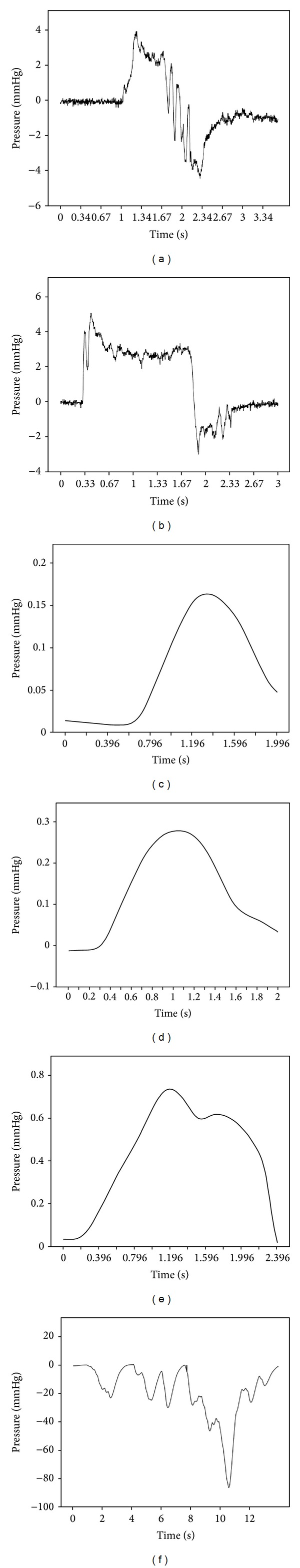
(a) Biphasic pressure change related to opening of the round window membrane with a needle. (b) Biphasic pressure change related to opening of the round window membrane with a cannula. (c) Unidirectional pressure change through opening of the round window membrane with a diode laser (4 W). (d) Unidirectional pressure change through opening of the round window membrane with a diode laser (6 W). (e) Unidirectional pressure change through opening of the round window membrane with a diode laser (10 W). (f) Unidirectional pressure change through opening of the round window membrane with a CO_2_ laser (20 W).

When opening the round window membrane with a needle, the maximum intracochlear pressure ranged from 1.59 to 4.59 mmHg, with a median of 3.28 mmHg (standard deviation (SD) ± 1.28). The course of the pressure change was recorded and depicted in real time in a graph. Furthermore, the velocity of the pressure gain was calculated (Table 2). For openings performed using a needle, the pressure gain velocity varied from 0.002 mmHg/msec to 0.05 mmHg/msec, with a median of 0.0176 mmHg/msec (SD ± 0.22). Openings using a cannula showed a maximum pressure gain from 1.51 mmHg to 5.06 mmHg, with a median of 3.84 mmHg (SD ± 1.45). The pressure gain velocity varied from 0.003 mmHg/msec to 0.04 mmHg/msec, with a median of 0.0666 mmHg/msec (SD ± 0.98). Comparison of the two groups using the *t*-test showed no statistically significant difference either for the maximum pressure gain (*P* > 0.05) or for the pressure gain velocity (*P* > 0.05). Both groups showed a biphasic pattern of pressure change related to opening (Figures 3(a) and 3(b)).

Opening of the round window membrane using a diode laser showed lower maximum pressure levels. Measurements with the diode laser were performed with different intensities. With an intensity of 4 W, the maximum pressure level ranged from 0.14 mmHg to 0.63 mmHg, with a median of 0.31 mmHg (SD ± 0.22). Slightly higher values were measured using the diode laser at 6 W. Values for the maximum pressure ranged from 0.15 mmHg to 0.88 mmHg, with a median of 0.29 mmHg (SD ± 0.27). Using 10 W, the maximum pressure values were higher, ranging from 0.39 mmHg to 2.18 mmHg, with a median of 1.08 mmHg (SD ± 0.31). The observed pattern was a unidirectional pressure change (Figures 3(c)–3(e)).

The paired *t*-test for the maximum pressure of the opening using a needle in comparison with a diode laser showed statistically significant differences. For all levels of intensity (4 W, 6 W, and 10 W) in comparison with the needle, a statistically significant difference (*P* < 0.05) was observed. Usage of a cannula in comparison with a diode laser also showed a statistically significant difference (*P* < 0.05) for every level of intensity (4 W, 6 W, and 10 W). Comparison of the maximum pressure values between the different groups of the diode laser showed no statistically significant difference (*t*-test, *P* > 0.05) for the intensity level of 4 W versus 6 W. However, comparing the rather low maximum pressure results for 4 W and 6 W with the intensity level of 10 W, a statistically significant difference (*t*-test, *P* = 0.044) was observed.

Regarding the pressure gain velocity, the lowest gain was found at 4 W. Using 4 W, the pressure gain velocity ranged from 0.001 mmHg/msec to 0.0005 mmHg/msec, with a median of 0.0003 mmHg/sec (SD ± 0.00015). Values using the diode laser at 6 W showed a higher pressure gain velocity, ranging from 0.00008 mmHg/msec to 0.003 mmHg/msec, with a median of 0.0009 mmHg/msec (SD ± 0.00122). Slightly lower values were observed with 10 W. In this group, the pressure gain velocity values ranged from 0.0001 mmHg/msec to 0.0007 mmHg/msec, with a median of 0.0004 mmHg/msec (SD ± 0.00026).

Statistical evaluation also showed a statistically significant difference for the pressure gain velocity. The paired *t*-test showed a statistically significant difference (*P* < 0.05) for the pressure gain velocity of the needle and the diode laser at every level of intensity (4 W, 6 W, and 10 W). The difference between the higher pressure gain velocity of the cannula in comparison with the lower diode laser pressure gain velocity (at 4 W, 6 W, and 10 W) was statistically significant (*t*-test, *P* < 0.05). Within the different groups of the diode laser, no statistically significant difference was seen between the different intensity levels (*t*-test, *P* > 0.05).

In contrast to the openings with the other tools described above, opening of the round window membrane using a CO_2_ laser showed a negative and high amplitude pressure direction (Figure 3(f)). The intracochlear pressure decreased from −0.24 mmHg to −50.16 mmHg, with a median of −20.1 mmHg (SD ± 19.81). However, the pressure gain velocity was rather low. It ranged from 0.0003 mmHg/msec to 0.011 mmHg/msec, with a median of 0.005 mmHg/sec (SD ± 0.00553). Opening of the round window membrane using a CO_2_ laser was rather difficult as it took obviously longer to perforate the membrane in comparison with the other tools. This long opening period was reflected in the low pressure gain velocity.

## 4. Discussion

The criteria for cochlea implantation have changed over the past years. The preservation of residual hearing has become one of the goals in modern cochlea implantation [2, 5, 9, 13, 14]. Preservation of residual acoustic hearing is closely connected with an atraumatic CI electrode insertion. Access to the cochlea via the round window is a widely used access for an atraumatic CI electrode insertion [4, 6, 15]. The opening procedure of the round window membrane itself is less frequently described in the literature. Briggs et al. [16] used a hypodermic needle in their temporal bone study to open the round window membrane after using a 1 mm diamond burr for a full exposure of the round window membrane [16].

**Figure 4:**
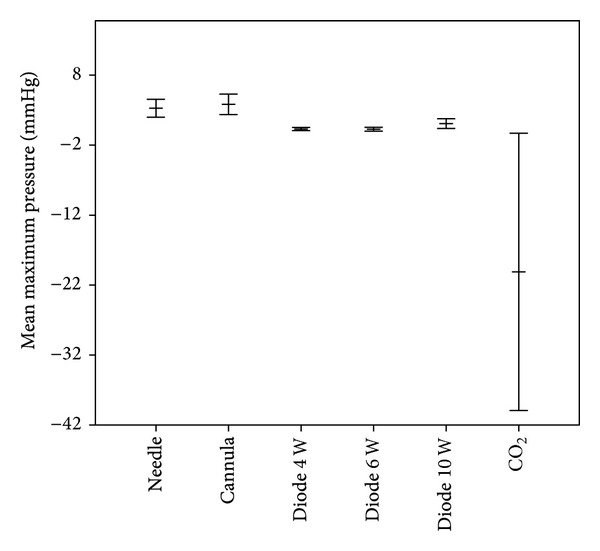
Mean maximum pressure values for opening of the round window membrane with standard deviation.

**Figure 5:**
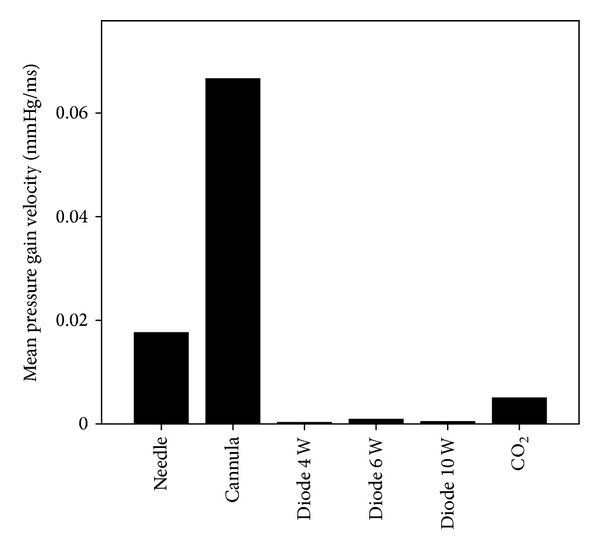
Mean pressure gain velocity for opening of the round window membrane.

In our study, we compared the opening of the round window membrane using different tools with regard to the intracochlear pressure change. In every experiment, it was ensured that air was not captured within the cochlea and that the sensor tip was completely sealed to the cochlea model. Mechanical opening of the round window membrane using a needle or sharp cannula showed no significant difference either in the maximum pressure value or in the pressure gain velocity (Figures 4 and 5). Whilst mechanical opening was attended by a rather fast pressure gain and rather high maximum pressure values, opening with a diode laser showed significant lower maximum pressure values and a lower pressure gain velocity (Figures 4 and 5). A less traumatic opening with the diode laser in vivo can be assumed. In contrast to the needle, cannula, and the diode laser, opening of the round window membrane with a CO_2_ laser caused negative pressure, with high negative pressure maximum values.

After opening the round window membrane with a CO_2_ laser, the fluidity within the cochlea was reduced and air bubbles were observed just underneath the round window membrane. As fluid loss was not observed, it can be assumed that the fluid had evaporated.

We could prove that even opening of the round window membrane leads to intracochlear pressure variation. For the surgeon, one of the main goals is to minimise intracochlear trauma. The influence of the intracochlear pressure variation on intracochlear trauma remains unclear. It should be presumed that less intracochlear pressure leads to less intracochlear trauma. Regarding the probability of intracochlear trauma related to pressure changes, hydrostatic, slow fluid pressure changes have to be separated from fast sound pressure-related fluid pressure changes. The literature related to this topic is limited and does not offer clear answers [17, 18].

Sound induced intracochlear pressure changes are widely described in the literature. In a gerbils model a maximum of 10 Pa was measured in scala vestibuli with a stimulus of 90 dB SPL at 15 kHz in the ear channel [19]. In scala tympani, 3.5 mm from the stapes with an input of 80 dB SPL at the stapes, the pressure varies up to 90 dB SPL (0.63 Pa) near the basilar membrane [20]. Our data from mechanical round window membrane openings show hydrostatic pressure changes up to 5.06 mmHg (675 Pa) and laser diode data up to 2.18 mmHg (291 Pa). In contrast to sound induced intracochlear pressure changes, manipulation to the cochlea seems to cause multiple greater pressure shifts. How hydrostatic pressure changes are conferrable and comparable to sound induced pressure changes needs to be investigated furthermore.

The approach taken in our study has some limitations regarding the applicability to the human cochlea. It has to be considered that the intracochlear hydrostatic pressure changes in vivo and in temporal bone measurements due to natural drain systems. The cochlea model was sealed with fibrin glue and fluid loss was avoided; it was therefore a sealed system with only one channel of fluid drain, the assumed round window. The human cochlea and the vestibule are a functional unit. Fluid pressure transfer between the different labyrinthine compartments is widely described [21–23]. Therefore, a direct transfer to the saccus endolymphaticus as a pressure equalisation unit could be speculated. However, this must be assumed to be limited in vivo. A similar limited quantity of fluid pressure transfer can be assumed for the aqueductus cochlea in a regular cochlea [24]. Surgical experience in a normal cochlea has shown that there is no or only a limited pressure load on the cochlea, which overcomes surface tension pressure, since we have observed no or only a limited degree of fluid outflow if the round window is opened during CI surgery [25].

Regular intracranial pressure is known to be age-dependent and ranges from 5 mmHg in young patients to 15 mmHg in old patients [26]. Because of a lack of regular human data, we have to compare the observed values with animal data or the clinical experience we have gained from cochlea implantation in anomalous cochlea with so-called gushers of intense endolymphatic outflow [25].

Since regular human intracochlear pressure data are missing, the valve capacity of the borderline structures between cochlea and intracranial pressures remains unclear and a transfer of the measured model values into in vivo behaviour is problematic. The estimation of pattern and principles of fluid pressure changes related to manual or mechanic handlings in terms cochlea implantation is essential for the establishment of a reproducible atraumatic cochlea implantation. The observed differences in opening the cochlea underline the importance of this specific substep of electrode implantation.

## 5. Conclusion

We provide the first results of fluid pressure changes due to the opening of an artificial round window membrane. To transfer the results to the human cochlea temporal bone, more experiments are needed. But the differences in the used model underline the possible importance of how to open the round window for an atraumatic cochlea implant procedure.

## Figures and Tables

**Table 1:** Maximum intracochlear pressure in mmHg while opening the round window membrane (five measurements).

	1	2	3	4	5	SD±
Needle	1.591003418	3.922973633	4.591782227	3.394470215	2.554870605	1.28
Cannula	1.505615234	4.578796387	4.7109375	5.056640625	3.363525391	1.45
Diode 4 W	0.165161133	0.163818359	0.440979004	0.633850098	0.135986328	0.22
Diode 6 W	0.75402832	0.27911377	0.161071777	0.088500977	0.147033691	0.27
Diode 10 W	0.80480957	0.39440918	1.293884277	0.73626709	2.176696777	0.31
CO2 10 W	−0.235107422	−4.120239258	−22.76672363	−50.15893555	−23.21478271	19.81

**Table 2:** Pressure gain velocity in mmHg/msec (five measurements).

	1	2	3	4	5	SD±
Needle	0.002	0.002	0.032	0.05	0.002	0.22
Cannula	0.02	0.003	0.03	0.04	0.24	0.98
Diode 4 W	0.0002	0.0002	0.0003	0.0005	0.0001	0.00015
Diode 6 W	0.0008	0.003	0.0003	0.00008	0.0002	0.00122
Diode 10 W	0.0003	0.0004	0.0001	0.0007	0.0007	0.00026
CO2 10 W	0.0003	0.0006	0.011	0.011	0.002	0.00553
